# In-Situ Li-Ion Pouch Cell Diagnostics Utilising Plasmonic Based Optical Fibre Sensors

**DOI:** 10.3390/s22030738

**Published:** 2022-01-19

**Authors:** Christopher Gardner, Elin Langhammer, Wenjia Du, Dan J. L. Brett, Paul R. Shearing, Alexander J. Roberts, Tazdin Amietszajew

**Affiliations:** 1 Centre for Advanced Low Carbon Propulsion Systems, Institute for Clean Growth and Future Mobility, Coventry University, Coventry CV1 5FB, UK; alexander.roberts@coventry.ac.uk (A.J.R.); taz.amietszajew@coventry.ac.uk (T.A.); 2 Insplorion AB, Arvid Wallgrens Backe 20, 413 46 Goteborg, Sweden; elin.langhammer@insplorion.com; 3 Electrochemical Innovation Lab, Department of Chemical Engineering, University College London, London WC1E 6BT, UK; wenjia.du@ucl.ac.uk (W.D.); d.brett@ucl.ac.uk (D.J.L.B.); p.shearing@ucl.ac.uk (P.R.S.); 4 The Faraday Institution, Quad One, Harwell Science and Innovation Campus, Didcot OX11 0RA, UK

**Keywords:** Li-Ion battery, pouch cells, battery, diagnostics, plasmonic sensing, optical fibres

## Abstract

As the drive to improve the cost, performance characteristics and safety of lithium-ion batteries increases with adoption, one area where significant value could be added is that of battery diagnostics. This paper documents an investigation into the use of plasmonic-based optical fibre sensors, inserted internally into 1.4 Ah lithium-ion pouch cells, as a real time and in-situ diagnostic technique. The successful implementation of the fibres inside pouch cells is detailed and promising correlation with battery state is reported, while having negligible impact on cell performance in terms of capacity and columbic efficiency. The testing carried out includes standard cycling and galvanostatic intermittent titration technique (GITT) tests, and the use of a reference electrode to correlate with the anode and cathode readings separately. Further observations are made around the sensor and analyte interaction mechanisms, robustness of sensors and suggested further developments. These finding show that a plasmonic-based optical fibre sensor may have potential as an opto-electrochemical diagnostic technique for lithium-ion batteries, offering an unprecedented view into internal cell phenomena.

## 1. Introduction

The lithium-ion battery market has grown dramatically over the past decades, driven by their widespread adoption in portable electronic devices (PEDs), automotive and grid storage sectors. Forecasts vary but are consistent in predicating significant further growth in lithium-ion production, driven primarily by the increasing adoption of electric vehicles (EVs) in the automotive industry, but also in other applications such as portable electronic devices, grid storage, micro-grids, aerospace and space [1,2,3,4].

Incremental improvements in battery technology have been and will continue to be vital in improving the viability, safety and environmental impact of lithium-ion batteries. In electric vehicles for example, batteries make up a significant proportion of the cost (up to a third), weight and volume of the vehicles, as well as having significant safety implications [5,6]. The charging time and range are also crucial vehicle attributes that are heavily influenced by the battery design [7]. Developments that improve these attributes will continue to drive EV adoption. With regards to environmental impact, efficient use of material, re-using (for example second life applications) and recycling batteries is crucial [8,9,10,11].

Battery diagnostics is seen as one area that can drive improvements in battery performance. Superior battery diagnostics could improve energy density of batteries and reduce the cost over lifecycle, by allowing superior battery state monitoring such that the battery can operate longer and closer to its limits. Improved battery diagnostics could also aid in safety monitoring through the prompt identification of failure mechanisms. Additionally, improved diagnostics enable easier re-use of batteries in second life applications due to the necessity to be able to evaluate the state of batteries that may have had dramatically different first life usages and degradation, as well as potentially supporting other approaches such as battery swapping (in lieu of in-situ charging) [12,13].

The dominant form of battery state tracking in applications today is voltage profiling and coulomb counting [14]. Coulomb counting accuracy is limited by such factors as the accuracy of the current measurement, the integration technique, the inability to account for internal losses in the battery, the open loop nature of technique causing the errors to accrue over time and the drift over time found in lower cost commercial current sensors [15]. While State-of-Charge (SOC) errors may only be in the region of 1% over a small number of cycles, or longer with well-developed models for specific battery packs, ultimately SOC errors of at least 3% are expected over time even in highly accurate coulomb counting models [14,16]. Alternative diagnostic techniques, such as open circuit voltage (OCV) measurements, electrochemical voltage spectroscopy (EVS) and impedance measurements tend to be more qualitative in nature unless in very controlled conditions with an extensive amount of testing [17,18,19].

In the area of real time and in-situ battery diagnostics there are a variety of techniques at an early technology level. An ideal in-situ diagnostic method would be one which can be installed without requiring a specially modified cell, that does not have a difficult cell manufacturing or assembly process, that does not impact the performance or durability of the cell, that provides the maximum amount of electrochemical data and is low cost. Existing techniques include reference electrodes which are used extensively in research to provide voltage information for both electrodes, but are not generally used in production applications due to technical limitations, manufacturing challenges, potential impact on cell performance and cost [20,21,22]. Thermistors can be used to measure temperature, which can provide important information about battery operating limits and safety, but do not correlate to SOC or State-of-Health (SOH) [22,23]. X-ray computed tomography has been demonstrated as a technique that can be utilised to detect microscale defects in cells [24], while ultrasonic techniques have been shown to correlate signal wave height with battery cell SoC [25]. Externally applied strain gauges have been used to monitor expansion of a cell and correlate it to cell degradation [26] and a variation on this setup has been shown to measure the volumetric expansion of a cell associated with SEI layer growth [27].

In terms of fibre optic battery diagnostics, extensive work has been carried out with Fibre Bragg Gratings (FBG) and Tilted Fibre Bragg Gratings (TFBG). Studies have demonstrated the capability of these devices to measure battery temperature and strain externally [28] and internally [22,29,30], with various techniques for deconvolution allowing highly accurate independent temperature and strain measurements [31]. Potential links between strain and battery cell SoC have been hypothesised and in certain conditions a highly accurate relationship between internally located FBG strain measurement and battery SoC has been demonstrated over a number of cycles [32]. Optical fibres utilising attenuated total reflection have shown a potential relationship between battery state and the corresponding optical signal, but with limited correlation and accuracy [33]. Colorimetry has also been shown to indicate battery state by measuring the cathode colour as it changes with lithiation, in a modified cell with a glass window [34].

In an attempt to expand available SoC or SoH correlatable in-situ diagnostics techniques, this paper documents the use of a plasmonic sensor, detailed further in the methodology section, coupled to an optical fibre inside a lithium-ion pouch cell. Plasmonic sensing is currently most notably and extensively used in bio-technology for biomelecular study and detection, as well as nanomaterial and catalyst study, in-situ and in operando [35,36]; developments in plasmonic sensor designs and bimolecular applications continue apace [37,38]. The technique measures changes in the refractive index of the adjacent substance via the impact that has on the resonant frequency of the surface plasmon waves (SPW) of a metal coating such as gold; in turn this is measured through internally reflected light intensity loss at the frequency of the SPW [35,36,39,40,41]. The technology presents itself as a technology with numerous advantages applicable to lithium-ion batteries, namely the potential for in-situ and continuous monitoring of electrochemical activity (the electrode potential and state of charge), the compact size, flexibility, chemical robustness, low cost of sensors and remote operating capability [35]. This method could potentially provide electrochemical data that none of the aforementioned existing in-situ methods can provide, without impacting performance of the cell or needing to adapt the cell to accommodate the diagnostic method.

The benefits and hence motivation of developing a more accurate real time battery diagnostic technique can be envisaged. The battery could be run closer to its charge and discharge limits, allowing battery sizing to be closer to what is required for a given application, resulting in battery cost and weight savings. Battery charge profile impacts on batteries could be better understood, allowing further reduction of the recharge times by developing more optimised fast-charge regimes. Second life applications could also be better enabled, as a key challenge around retired batteries is developing a reliable and efficient method of measuring the current State of Health. This could be of particular benefit in the EV industry as predictions of up to 120 GWh of retired batteries with up to 80% capacity remaining by 2028 gives an idea of the scale of the second life reuse potential [8].

The Battery Management System, crucial in managing cell activity in battery modules and packs through voltage and current controls, relies on cell data to ensure safe operation, e.g., by avoiding overcharge [3]. The potential for plasmonic sensing to provide real time data, or a periodic reference for the BMS to re-calibrate according to the current state, coupled with superior accuracy by taking measurements from inside the cell, opens up the possibility for the BMS to significantly optimise the use of the battery cells with reduced error and better safety margins. Improving battery management through superior diagnostics can also benefit reliability and lifespan [42].

The application of plasmonic sensing technologies in energy storage devices is novel. There has been an investigation into the use of the technology in a super-capacitor, with promising findings [43]. In terms of batteries, the only reports the author is aware of is a preliminary investigation into a single layer lithium iron phosphate battery pouch cell battery in early 2021 [44,45]. This investigation utilises plasmonic sensing technology inside prototype 1400 mAh lithium-ion nickel manganese cobalt (NMC) versus graphite pouch cells. We report successful implementation, correlation of the sensor reading with battery state and minimal impact on the cell performance.

## 2. Experimental Methodology

### 2.1. Plasmonic Sensor Technology

**Figure 1:**
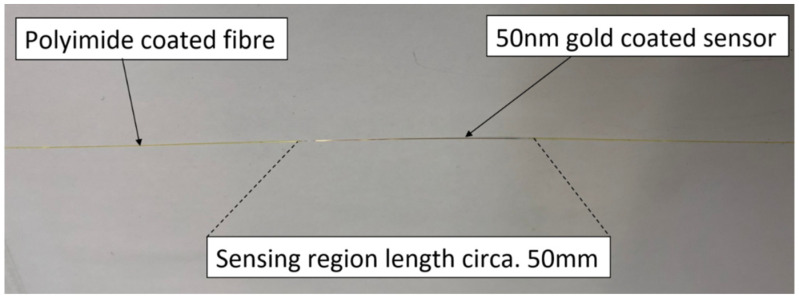
Annotated picture of the optical fibre plasmonic sensor.

The optical measurements in the experimentation are carried out utilising plasmonic sensor fibres and an optical unit transmitter and receiver device (provided by Insplorion AB, Göteborg, Sweden). The fibres used are optical fibres with a 50 mm sensing region coated in a thin gold film, the fibre design is illustrated in Figure 1. The sensing region and rest of the fibre have polymer coatings, including polyamide as a proven protective coating inside cells, to protect it from corrosion within the battery cell and improve mechanical robustness.

The optical unit consists of a broadband light emitter and an optical spectrometer receiver, the sensor fibre is used in transmission mode-transmitter at one end and the receiver at the other. The sensor fibre is fusion spliced to optical fibre ‘pigtails’ connected to the optical unit via FC/PC connectors. The fibre optic cables and ‘pigtails’ connecting the sensor to the optical unit are low hydroxyl multimode Ø105 µm silica fibre cores (Thorlabs). A plasmonic response causes high attenuation at a light wavelength corresponding to the resonant frequency, influenced by the refractive index of the analyte-this relationship leads to an optical response that corresponds to the surrounding analyte change [46]. In this experimentation we are testing if an analyte change in the battery that correlates to battery state can be measured by the sensor; one such potential change is lithium concentration at the electrode.

The optical unit (OU) used is provided by Insplorion AB (Göteborg, Sweden) including software for control of the optical unit settings and data capture. The integration time and number of measurement averages taken per measurement are selected to ensure an accurate reading without saturating the optical receiver. The sampling rate typically varies between once every 60 s and 120 s depending on the length and nature of the test, to ensure sufficient resolution.

### 2.2. Testing Methodology

Testing carried out will be divided into two sections, sensor testing in solvent and electrolyte solutions and sensor testing inside pouch cells. The sensors were tested in electrolyte solutions to potentially identify plasmonic responses and wavelengths of interest. In parallel work was undertaken to assemble the fibre optic sensors inside pouch cells, allowing the cycling and measurement of battery cells while simultaneously measuring the optical signal response.

### 2.3. Electrolyte Solution Analysis

The spectrometer connected to the optical sensor emits broadband light across the wavelengths 400 nm to 1000 nm, a range selected for compatibility with the receiver and the plasmonic sensor [44]. In order to identify which wavelengths from the range may be of particular interest and most likely to exhibit a plasmonic response inside a battery cell, the sensor has been tested in battery cell electrolyte and then in pure organic solvent with increasing molar concentrations of lithium hexafluorophosphate (LiPH_6_) salt, commonly used in battery electrolytes.

**Figure 2:**
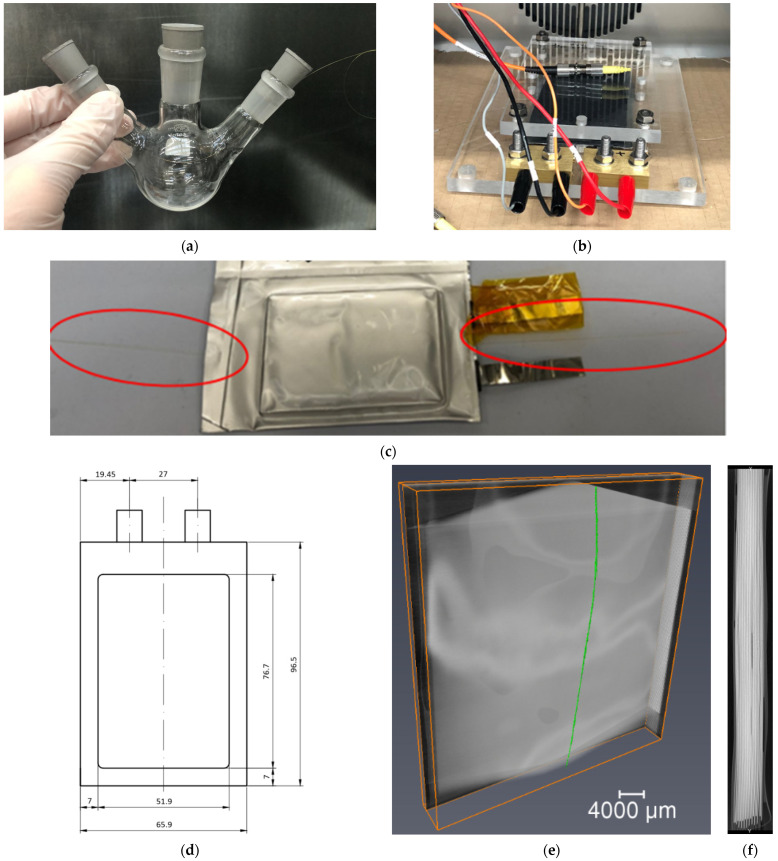
(**a**) Fibre sensor in 3 neck glass flask, (**b**) Sealed cell with sensor in jig connected to OU and potentiostat, (**c**) Fibre in sealed pouch cell, (**d**) Schematic of pouch cell dimensions (mm), (**e**) X-ray of fibre in cell, (**f**) Cell stack X-ray side profile (5 mm width).

Plasmonic optical fibre sensors were initially placed in battery cell electrolyte in an attempt to identify the plasmonic response to the solution and the effect of extended exposure to electrolyte. A 3-neck round bottom flask was used with the optical fibre sensor placed into the flask through the side necks. Bungs were placed in each side neck to hold the fibres in place and seal the vessel. The complete setup can be seen in Figure 2. A reference reading in air was taken first, subsequently the vessel was then placed inside an Argon atmosphere glovebox where electrolyte was poured into the vessel with a pipette through the central neck. 15 mL of 1 M Lithium hexafluorophosphate (LiPF6) in Ethylene Carbonate/Ethyl Methyl Carbonate (EC/EMC-3/7 *v*/*v*) +2% weight Vinylene Carbonate (VC) electrolyte was added, submerging the sensor regions of the fibres. The sensors were then plugged into the optical unit and the spectra readings were recorded; this procedure enabled an optical response comparison between the electrolyte and an air reference.

Subsequently, to de-convolute the impact of the lithium salt on the OU response from that of the organic solvent itself, an empty 3-neck round bottom flask was placed inside a glovebox, where pure organic solvent (Dimethyl carbonate—DMC) was first added to the flask to obtain a solvent only optical response. Subsequently lithium salt electrolyte (1 M LiPF6 in EC/EMC (3/7 *v*/*v*)) was added to observe how the signal changes in relation to incrementally increasing lithium salt concentration, up to 0.5 molar concentration of LiPF6.

### 2.4. Cell Manufacture and Sensor Integration

In terms of sensor in cell testing, pouch cell technology is selected for this experimentation for its relatively flexible manufacturing process conducive to accommodating sensors. A method has been developed to reproducibly manufacture pouch cells with fibre optic sensors inside, suitable for the production of small batches of prototype cells. The pouch cells used weigh 29.4 g +/− 0.2 g when dry (no electrolyte), with lithium nickel manganese cobalt oxide (LiNiMnCoO_2_, NMC 111) cathode and graphite anode at a loading of 2 mAh cm^−2^.

The optical fibre sensor was introduced through the electrode stack along the vertical axis centred in parallel between the two cell tabs, adjacent to the anode three electrodes in from the edge of the stack, prior to sealing the cell. This location was chosen as the centre of an electrode a few layers in is likely to be more representative of the overall cell state than an edge case. Additionally, as the sensing region is 50 mm in length means it should take an average of the behaviour over that length. The plasmonic sensor has a shallow sensing range, approximately 1000 nm [36,47], so the position of the sensor should mean it only detects the electrode immediately adjacent to it.

Once the fibre was placed in position the first pouch cell seals along the top and bottom of the cell were made, holding the fibre in place. Subsequently the cell was filled with electrolyte inside an argon atmosphere glovebox and the final seal made under vacuum. The cells are filled with 9.5 g (8.6 mL) of electrolyte 1 M LiPF6 in EC/EMC (3/7 *v*/*v*). The manufactured cells have an approximate capacity of 1400 mAh. This results in the completed cell with integrated fibre sensor, as can be seen in Figure 2c.

The cells with fibre sensors were prepared for testing which will be described in greater detail in the subsequent sections. In addition to this, three reference cells without fibres in were prepared, to allow comparison of the battery cell performance with and without the sensors and evaluate the impact of the sensor on cell performance. Finally, one of the cells with fibre sensors also includes a pure lithium reference electrode, to allow evaluation of the cathode and anode voltage separately with respect to the optical signal.

### 2.5. Lab-Based X-ray Tomographic Imaging of Cell

High energy lab-based X-ray imaging of one of the 1.4 Ah pouch cells was performed on a Nikon XTH 225 instrument (Nikon Metrology, Tring, UK) using a W target and a 1 mm Cu filter. The scan of the cell with internal fibre sensor was carried out using a voltage of 160 kV and a total power of 12.8 W. For CT dataset, 3185 projections were obtained with an exposure time of 1 s to minimize the artefacts. The fibre was maintained within the field of view (FOV) to obtain a spatial resolution with voxel size of ca.15 µm. Reconstruction of obtained projections was conducted using Nikon CT Pro 3D software (Version XT 4.4.4, Nikon Metrology, Tring, UK) with three-dimensional visualisation of dataset being performed using Avizo 2019. 4 (FEI, Lyon, France). An X-ray image in Figure 2e shows the position of the fibre within the cell as well as the electrode stacks from the side view.

### 2.6. Cell Formation and Test Cycling

The pouch cells were manufactured with the plasmonic sensors adjacent to the anode, with the intent of enabling measurement of lithium concentration at the electrodes. The sensing fibre is spliced to pigtail fibres with FC/PC connectors and connected to the optical unit’s broadband light transmitter and receiver. The cells are placed inside a plastic jig for compression with a foam separator, to maintain the structural integrity of the cell during cycling. The tabs of the cell are then connected to multi-channel potentiostats (VMP3, BioLogic, Seyssinet-Pariset, France) in a 4-wire configuration. Figure 2b shows fibre-instrumented cell mounted in a test jig under mild compression, connected to the optical and potentiostat test equipment. The simultaneous collection of optical and electrochemical data allows for the evaluation and correlation between the battery state and the optical signal response.

All cells were formed using two cycles of constant current charge at C/20 to 4.2 V, with constant voltage to a current of C/100 with subsequent discharge to 2.5 V at C/20. Subsequently the majority of the testing was conducted under charge and discharge cycles at a C/5 rate, a galvanostatic intermittent titration technique test was also conducted. The test program carried out on the cells is detailed in Table 1.

### 2.7. Autopsy of Cycled Cells

Cell dissection and analysis of cycled cells has been carried out to observe the impact of the presence of the sensor fibre on the cell, and the condition of the sensor after being inside a cycled cell. The cell pouch is cut open with a scalpel and the first two electrodes and separator layers removed to get to the third electrode with the adjacent fibre sensor. Samples of the electrode are subsequently taken for microscopic analysis; the morphology of the anode samples are studied using scanning electron microscopy (SEM) (ZEISS Sigma 500, Zeiss, Oberkochen, Germany) at an electron high tension (EHT) voltage of 10 kV, using a secondary electron detector.

## 3. Results and Discussion

The optical fibre sensors were successfully implemented and tested. Data collected from the sensor in electrolyte solution and sensor in cell testing, as described in previous sections, has been processed to observe correlation and any defining features. The results are divided into sensor in electrolyte solution, sensors in cells on slow charge/discharge cycle, GITT testing, impact on cell and sensor performance and cell autopsy.

### 3.1. Fibre Optic Plasmonic Sensor in Solvent and Electrolyte Solutions

**Figure 3:**
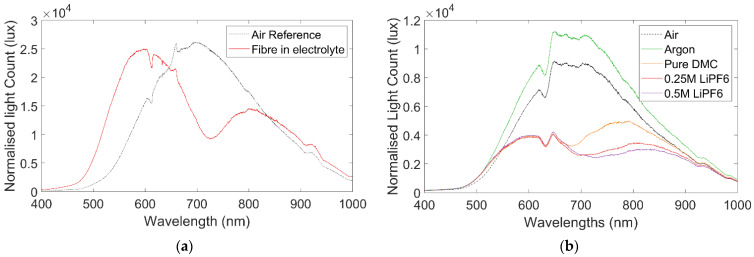
(**a**) Spectra reading in EC/EMC and 1 M LiPF6 salt electrolyte, (**b**) Spectra readings of fibre in solvent and incrementally increasing LiPF6 concentration to 0.5 M. Clear plasmonic response around 725 nm can be observed in both cases.

The optical readings response of the plasmonic sensor to air and electrolyte is illustrated in Figure 3a, the significant increase in light extinction is observed in the 725 nm region (represented as a trough in the light count). This indicates that the strongest plasmonic response to the lithium salt electrolyte solution is in that region; it is expected that this is due to the overall refractive index of the solution, rather than any particular element or compound adjacent to the surface of the sensor.

A further experiment in which electrolyte was incrementally added to a pure solvent, shown in Figure 3b, clarified this to some degree. The sensor reading in the pure solvent indicated a limited plasmonic response in the region of 680 nm. However, as the electrolyte with lithium salt was added to the mixture the profile of the OU response began to change, creating a significantly stronger plasmonic response in the 725 nm region again. Making the reasonable assumption of homogenous solution distribution, this implies the refractive index of the pure solvent elicits a plasmonic response in the 680 nm region, while increasing the lithium salt concentration alters the refractive index such that the wavelength of the plasmonic response increases. As such, in testing utilising the sensors inside active cells, the light count of the wavelengths in the region around 725 nm are considered of particular interest; this conclusion is supported by a similar study conducted into utilisation of plasmonic sensors in prototype cells [44].

### 3.2. Fibre Optic Plasmonic Sensor in Cell Cycling

**Figure 4:**
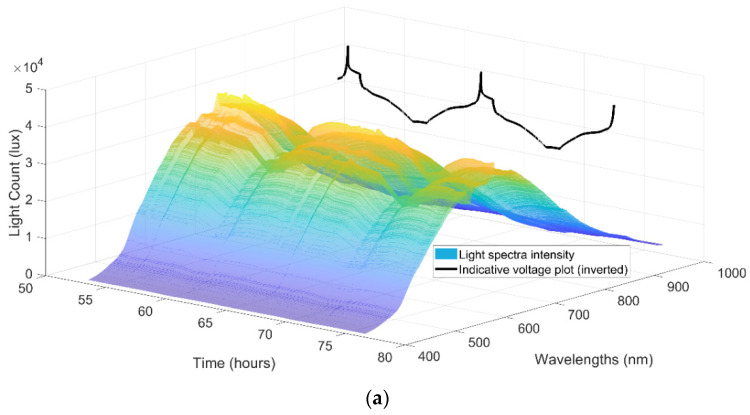

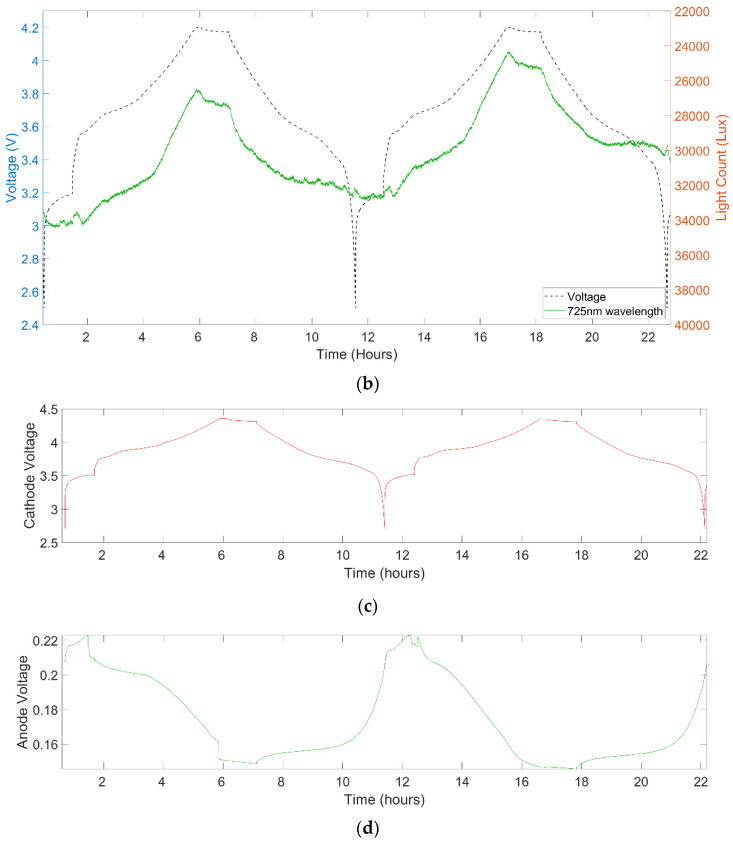
(**a**) Light intensity spectra over two cycles, (**b**) Cell voltage and 725 nm light count over two cycles, (**c**) Referenced cell cathode voltage and (**d**) Referenced cell anode voltage.

The optical signal response displayed a clear correlation with voltage during cell cycling, as illustrated in Figure 4, which represents the comprehensive broadband optical signal response over two cycles. The correlation is most discernible in the 700 nm wavelength region, while a small degree of correlation can be observed across the entire 400 nm to 1000 nm spectrum.

Focusing specifically on the 725 nm wavelength noted earlier, a clear correlation can be seen in Figure 4b between the optical signal and the measured cell voltage. This correlation is strongest for the parts of the cycle above a full cell voltage of approximately 3.6 V, where the profile very clearly matches, while the profile correlates more loosely at the lower voltage regions of the cycle, irrespective of the Ohmic potential drop. Notably 3.5 to 3.6 V is the voltage at which lithiation starts to occur in NMC versus graphite Li-Ion batteries, which supports the hypothesis of the optical response related to anode lithiation. Figure 4c,d display the cathode and anode voltages respectively, obtained using the implanted reference electrode, allowing observation of the optical signal relative to the separate electrode voltage profiles. Whether the light signal correlation is stronger with the anode, cathode or full cell voltage is not immediately obvious visually, although it is noted that light intensity change is positively correlated with the anode voltage change but inversely correlated with the cathode and full cell voltage change.

The mechanism by which the optical signal correlates to the cell voltage is hypothesised to be the sensor responding to the changing lithium concentrations at the surface of the anode, supported by the correlation of the impact on the optical sensor signal and the state of lithiation with increasing cell voltage/dropping anode voltage. While there is a clear correlation between the optical response and the electrochemical data, other variables that could impact the sensors include temperature, strain and pressure; another study has previously demonstrated that the sensor fibre signal is also sensitive to cell temperature and pressure [45]. The complete signal response can therefore be a combination of effects in addition to the primary plasmonic response herein evaluated; deconvolution of measurands will be the subject of further work to enable wider application.

### 3.3. GITT Cycling

**Figure 5:**
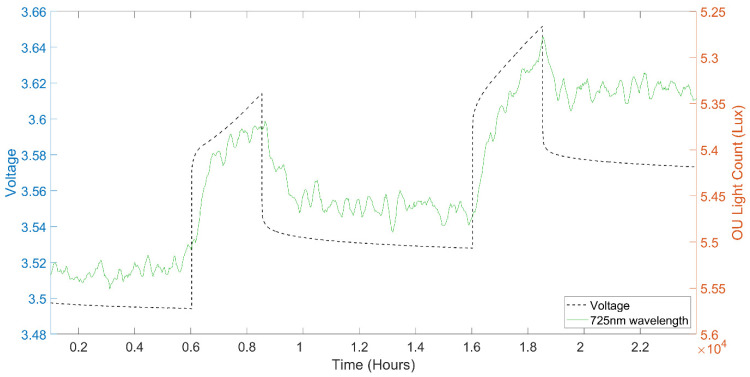
Cell voltage and OU response over two GITT cycles.

GITT cycling was used to observe the reaction of the signal to the voltage pulses, to further evaluate the correlation and relationship between cell state and optical signal. Figure 5 shows the 725 nm wavelength optical response over two representative pulses. The OU signal has been filtered with a Savitzky–Golay filter to reduce noise without distorting signal tendency.

The light extinction increases as voltage and charge increase, but does so with a steady and lagging response compared to the voltage increase. This indicates the signal does not directly correlate to the applied and measured cell voltage, and is more likely to correlate to a secondary effect of the applied voltage. Subsequently when the cell is at rest such that the charge is not increasing and the voltage relaxes as the lithium diffuses more equally into the anode, we see a relaxation in the optical signal with light extinction decreasing. This in turn demonstrates that the OU signal is not directly correlated to charge voltage and is potentially responding to secondary electro-chemical effects within the cell.

A potential mechanism that could explain this response is that the sensor is measuring or responding to lithium concentration at the surface of the anode. As the cell is charged, the lithium concentration at the anode surface is increasing, while when the cell is relaxing at rest the lithium is diffusing around the anode and the concentration decreases at the surface. This supports the previous conclusion about lithium concentration at the anode surface being the primary measurand.

### 3.4. Impact on Cell and Sensor Performance

**Figure 6:**
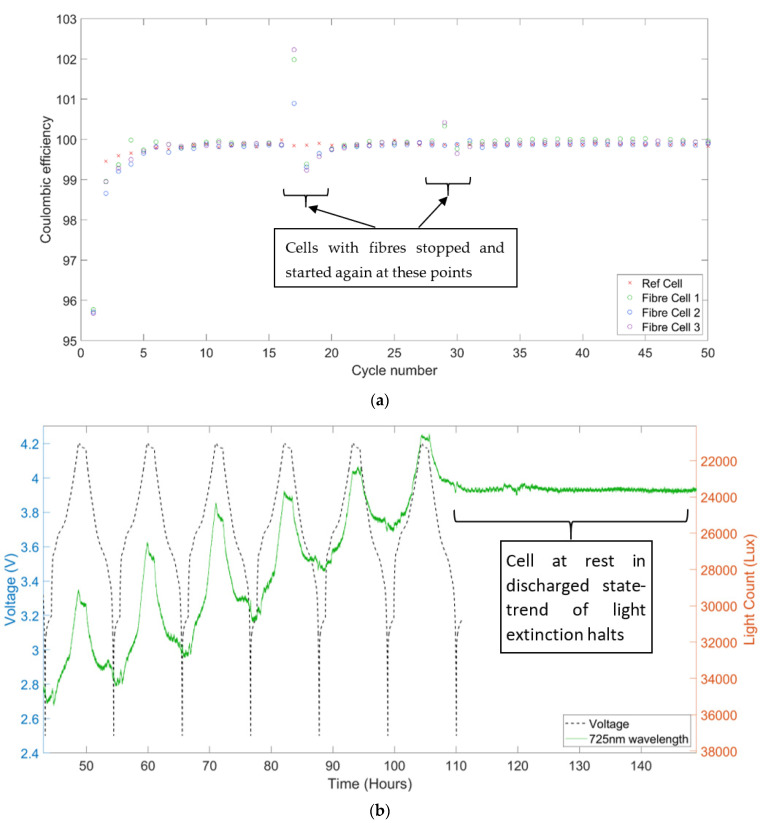
(**a**) Coulombic efficiencies of reference cell and cells with fibre sensors, (**b**) Battery cell voltage and OU light count.

Figure 6a shows the coulombic efficiencies of three fibre instrumented cells over 50 cycles, compared to a benchmark cell without the sensor. The data indicates that the presence of the sensor has little to no impact on the coulombic efficiency of the cell; this is significant, as an ideal diagnostic method should not interfere with the measured process, while high coulombic efficiency and long-term cycling are required for commercially relevant applications. The regions around the 16th and 28th cycles show some deviation in the cells with sensors coulombic efficiency; this is due to cell stop and restarts at those points to allow adjustment of the optical equipment integration time.

While the cell seems unaffected by the presence of the fibre sensor, Figure 6b illustrates that the fibre sensor signal deteriorates significantly as the cycling progresses, with light count dropping significantly over a number of cycles. The cell was subsequently cycled for a full 50 cycles, by the end of which the light intensity had declined asymptotically to towards zero.

The trend of increasing light extinction as the cell cycling progresses can be due to factors such as chemical corrosion of the sensor, internal battery changes such as SEI layer growth on the anode and/or around the fibre, or even potentially penetration and alloying of the lithium ions with the gold. Notably it can be seen in Figure 6b that the light extinction is clear while the cell is cycling but appears to stop when the cell is at rest after being discharged. This observation supports the hypothesis of electrode processes during cycling causing the signal loss; possible mechanisms include electrolyte decomposition and SEI growth around the sensor or lithium ion and gold alloying, linked to the higher voltage windows of the cycle.

### 3.5. Autopsy of Cycled Cells with Fibres

**Figure 7:**
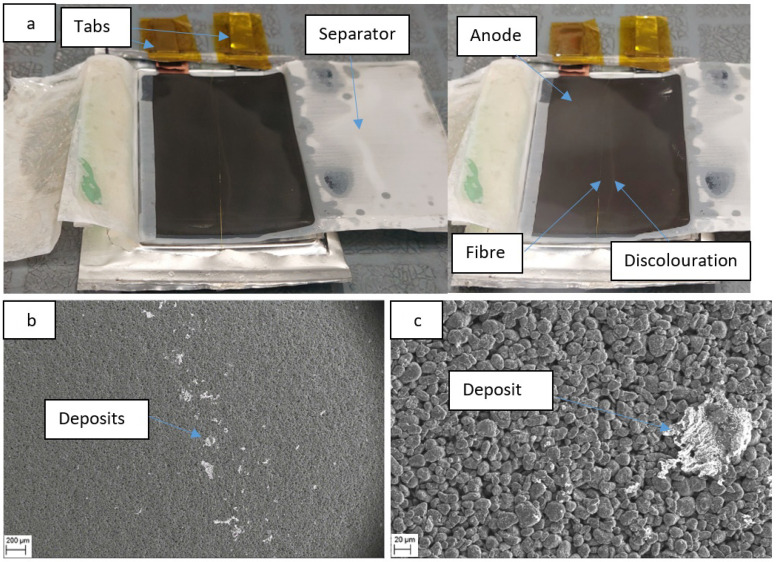
(**a**) Autopsied cell with sensor fibre, opened after 50 cycles, (**b**) SEM image of discoloured region on electrode at 73× magnification (**c**) SEM image at 566× magnification (enlarged section of figure (**b**).

Opening a cell that has been cycled for 50 cycles shows a discoloration on the surface of the electrode that follows the line of the fibre. This can be caused by ‘pooling’ of electrolyte around the fibre or uneven drying during cell disassembly. However, the coulombic efficiency data analysed earlier suggests that the presence of the fibre does not appear to impact cell performance. The sensor on the fibre also appears to have been delaminated or corroded in patches; this could be due to deposition or corrosion and requires further study, it may be the cause of the increasing light extinction trend shown in Figure 6b. The image of the opened cell can be seen in Figure 7a below.

SEM scans of an electrode sample from the centre of the anode, with part of the discolouration on, is shown in Figure 7b,c. The SEM images suggest that the line of lighter discolouration running down the electrode, apparently aligned to the fibre position, is due to large deposits on the electrode surface. These can be explained as lithium salt deposits appearing in a greater concentration in this area due to ‘pooling’ of electrolyte where the fibre sensor has pressed into the surface of the electrode.

## 4. Conclusions

In this study, we report a successful integration of plasmonic optical fibre sensor inside multiple pouch cell batteries, allowing the simultaneous measurement of battery state and optical signal response. The results demonstrate a clear, but not direct, correlation between battery cell voltage and optical signal light extinction. The cycling and GITT data indicates that the optical sensor readings can be correlated to battery state via secondary effects within the cell which we hypothesise to be related to anode surface lithiation. Furthermore, it has been demonstrated that the presence of the sensor has negligible impact on the performance of the cell itself with coulombic efficiencies of 99.8% being achieved during steady cycling up to 50 cycles.

These initial promising results suggest further investigation is warranted into the potential for this technology to determine in-situ, in operando battery state. Further data collection and modelling could enable the development of a tool that provides quantitative state of charge and state of health measurements, as well as rapid detection of battery failure modes such as lithium plating. This could potentially have applications ranging from providing additional information during testing of new battery types, to providing low cost and more effective battery diagnostics in commercial applications, to providing early detection of a battery failure event and improving battery safety.

Further work will include additional testing across different cycling profiles, testing with the sensor on the cathode and analysis of the data to quantitatively evaluate relationships between the OU signal and battery cell state of charge and health. Additionally, work should be undertaken to address the apparent deterioration of the sensor in the cell over time by evaluating sensor materials and protective coating options.

## Figures and Tables

**Table 1:** Cells test programs.

Test Type	Steps	Current/Voltage Input	Limit
50 charge and discharge cycles	Constant Current (CC) Charge	280 mA (C/5)	First of 4.2 V or 10 h
	Constant Voltage (CV) Charge Rest	4.2 V	First of 70 mA (C/20) or 2 h. 1 h
	Constant Current Discharge	280 mA (C/5)	First of 2.5 V or 10 h
	Repeat above (50 cycles)		
GITT	Constant Current (CC) Charge	280 mA (C/5)	15 min
	Rest	-	45 min
	Repeat steps (40 pulses)		4.2 V
	Constant Current (CC) Discharge	280 mA (C/5)	15 min
	Rest	-	45 min
	Repeat steps (40 pulses)		2.5 V

## Data Availability

The dataset generated and analysed during this study are available from the corresponding author on a reasonable request, but restrictions apply to the commercially confident details.
